# 

**Published:** 1850-10

**Authors:** 


### AMERICAN JOURNAL

OF

### DENTAL SCIENCE.

EDITED BY

CHAPIN A. HARRIS, M. D., D. D. S.

PROFESSOR OF THE PRINCIPLES AND PRACTICE OF DENTAL SURGERY IN THE BALTIMORE COLLEGE; MEMBER OF THE AMERICAN MEDICAL ASSOCIATION, ETC. ETC.;

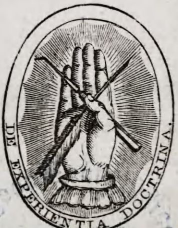

LIBRARY

21686

Washington, D.C.

NEW SERIES—VOLUME I.

PHILADELPHIA:
LINDSAY & BLAKISTON.
BALTIMORE:—ARMSTRONG & BERRY.
NEW ORLEANS:—JOHN BALL.
1850.—51.

W
1
AM 45 M

JOHN W. WOODS, PRINTER,
BALTIMORE.

We also announce, with regret, the decease of Dr. Lewis Roper, of Philadelphia. He died on his way from San Francisco, California, to Panama, of cholera.

We received a letter a few days since, announcing the death of Dr. J. Jones, of Dayton, O. Dr. Jones was not only highly esteemed as a dentist, but enjoyed the friendship and confidence of all with whom he was acquainted.

The last number of the Dental News Letter, announces, too, the death of Dr. Blakely, of Havana, Cuba.

### TO READERS AND CORRESPONDENTS.

Communications intended for publication should be directed to Dr. C. A. Harris, editor of the American Journal of Dental Science, Baltimore, Md. But all letters relating to the business of the Journal, or containing remittances in money, should be directed to Drs. C. A. Harris and A. A. Blandy, as the last named gentleman, who is associatad with Dr. H. in practice, will have the management of the *business* connected with the publication.

The following Journals and Publications have been received.

The American Journal of the Medical Sciences. Edited by Isaac Hays, M. D., July and August, 1850.

The London Medical Gazette, July, August and September, 1850.

The Boston Medical and Surgical. Edited by J. V. C. Smith, M. D. July, August and September, 1850.

The British and Foreign Medico-Chirurgical Review, July, 1850.

The New York Dental Recorder. Edited by C. C. Allen, M. D., July, August and September, 1850.

The Dental News Letter, July and October, 1850.

The London Medical Times, July, August and September, 1850.

The Provincial Medical and Surgical Journal. Edited by W. H. Rankin, M. D., and J. H. Walsh, Esq., June, July and August, 1850.

The Retrospect of Medicine, being a half-yearly Journal, containing a retrospective view of every discovery and practical improvement in the Medical Sciences. Edited by W. Braithwaite, M. D., January—June, 1850.

The Half-yearly Abstract of the Medical Sciences. Edited by H. Rankin, M. D., July, 1850.

The Dublin Medical Press, July, August and September, 1850.

British, American, Medical and Physical Journal. Edited by Archibald Hall, M. D. July, August and September, 1850.

The Medical Examiner. Edited by F. G. Smith, M. D., July, August, September and October, 1850.

Buffalo Medical Journal. Edited by Austin Flint, M. D.

The Charleston Medical Journal and Review. Edited by D. J. Cain, M. D. and F. Peyrl Porcher, M. D., July and September, 1850.

The New York Journal of Medicine, and the Collateral Sciences. Edited by S. S. Purple, M. D., July and September, 1850.

The Southern, Medical and Surgical Journal. Edited by J. P. Garvin, M. D., July, August, September and October, 1850.

The North-Western Medical and Surgical Journal. Edited by J. Evans, M. D., and E. G. Meek, M. D., July and August, 1850.

The New York Medical Gazette and Journal of Health. Edited by D. Meredith Reese, M. D., LL. D., July, August, September and October, 1850.

The Western Journal of Medicine and Surgery. Edited by L. P. Yandell, M. D., and T. S. Bell, M. D., July, August and September, 1850.

The St. Louis Medical and Surgical Journal. Edited by Drs. M. L. Linton, J. S. Moore, Wm. M. McPheeters, and J. B. Johnson. May and June, 1850.

The Ohio Medical and Surgical Journal. Edited by S. Hanbury Smith, M. D., July and September, 1850.

The Western Lancet and Hospital Reporter. Edited by L. M. Lawson, M. D., and George Mendenhall, M. D., July, August and September, 1850.

The New York Register of Medicine and Pharmacy. Edited by C. D. Griswold, M. D., Nos. 2 and 3, vol. 1.

The London Lancet, Journal of British and Foreign Medical, Surgical and Chemical Science, Criticism, Literature and News. Editor Thomas Wakley, M. D., for London; sub-editors J. H. Bennet, M. D., T. Wakley, M. R. C. S. E. July, August and September, 1850. From William Taylor & Co., Baltimore.

The Western Medical News. Edited by R. S. Newton, M. D., &c., and O. E. Newton, M. D., September, 1850.

Annual Announcement of Rush Medical College, Chicago, Illinois. Session 1850–'51.

Annual Announcement of Lectures of the Medical College of Ohio. For the Session of 1850–'51, and Catalogue of Graduates, for the Session of 1849–'50.

Annual Circular of the Medical Faculty of the Washington University of Baltimore. Session 1850–'51.

Sixth Annual Announcement of the Ohio College of Dental Surgery, for the Session 1850–'51.

Report of the Trial—The People versus Dr. Horatio N. Loomis, for Libel. Tried at the Erie County Oyer and Terminer, June 24, 1850.

Annual Announcement of the Medical Department of the St. Louis University. Session 1850–'51.

The Dental Messenger and Lancaster Annual Visitor, for 1849. By John McCalla, D. D. S., Lancaster, Pa.

Manual of Dentistry, containing a short History of the Teeth, their Diseases and Appropriate Remedies. By D. L. Stocking, M. D., St. Francesville, La.

Observations on the Teeth. By Dr. H. C. Wanzer, Rochester, N. Y. 1849.

We must decline publishing the communication from S. as it relates to a personal matter in which the members of the profession generally can feel no interest whatever. We will return the manuscript should he desire us to do so.

The report from D., although part of it is in type, is unavoidably excluded. It shall appear in our next number.

☞As the legal transfer of the Journal from the American Society of Dental Surgeons to the editor was not fully consummated until the twelfth of the present month, (November,) the publication of this number has necessarily been delayed considerably beyond the time when it should have been issued. Each future number will be issued the early part of the month in which it shall become due.

---

### CONTENTS.

### NEWS SHEET.

### American Journal of Dental Science,

EXTRA.

BALTIMORE, OCTOBER 1, 1850.

### PROSPECTUS

### OF A NEW SERIES OF THE

### AMERICAN JOURNAL OF DENTAL SCIENCE.

To the Members of the Dental Profession:

Gentlemen:—The American Society of Dental Surgeons having discontinued the publication of the American Journal and Library of Dental Science, the subscriber, impressed with the belief that the interests of the Profession require its continuance, has consented to assume the responsibility of its future publication. The Journal will, therefore, be henceforth under his immediate and exclusive control.

Past experience teaches that the task thus assumed will be no easy one, but the editor undertakes it with a resolute determination to perform the arduous duty to the very utmost of his ability; and relying with entire confidence upon the sympathy and aid of his professional brethren, he hopes to make the publication as indispensable to the Dentist, who may desire to keep pace with the progress of his art, as the best conducted Medical Journals are to the Surgeon and Physician.

In order to make the Journal more useful to its readers, the plan of the publication will be somewhat modified.

After the completion of the work on Dental Medicine, by Prof. Bond, now in course of publication, the Library part of the Journal will be discontinued, inasmuch as almost all the valuable European treatises upon Dental Surgery have already been republished in it.

The space heretofore devoted to the Library will be occupied by copious abstracts from all important papers or publications relating, directly or indirectly, to the Dental branch of Medicine, that shall appear in this country or in Europe.

The readers of the Journal will, in this way, be constantly informed of all discoveries or improvements which may, from time to time, be made in this department of the curative art.

The other part of the Journal will contain original communications, reviews, bibliographical and miscellaneous notices; and, in this connection, it may be proper to mention, that a number of the most eminent Dentists, both in this country and in Europe, have already kindly promised to contribute regularly to our pages; while others have consented to report all novel or peculiarly interesting cases and observations which they may think of sufficient importance to be communicated to the profession.

The editor respectfully solicits from his professional brethren, every where, similar oommunications and reports.

Regular European correspondents will be provided, who will give early information of all pertinent matters which may come under their notice.

A large number of the ablest Medical Journals are received in exchange, and will furnish much useful collateral matter.

With such facilities at his command, the editor hopes to be able to furnish a Periodical in every respect worthy of the present advanced and advancing state of Dental Surgery, and necessary to every scientific practitioner.

The first number of the new series will be issued the last of the present month. Gentlemen who may wish to receive it, are requested to signify the same by letter, post paid, to the editor, as early as possible, as it is not intended to print a much larger edition than shall be subscribed for.

The work will be printed upon superior paper, and with good type. Each number will contain, on an average, at least one hundred and sixty-eight pages, and the size will be increased as rapidly as the state of the subscription will warrant. As heretofore, it will be issued quarterly.

TERMS—Five dollars a year, in advance, or immediately after the receipt of the first number of each yearly volume.

Any one obtaining four subscribers to the American Journal of Dental Science, and remitting twenty dollars will receive a fifth copy gratis.

CHAPIN A. HARRIS.

---

### OUR NEWS SHEET.

It is the intention of the Publishers to issue twice a year, in connection with the American Journal of Dental Science, a News Sheet, which will contain all the advertisements of the former publication. A copy of this will be sent gratuitously to nearly every dentist in this country, as well as to a large number in Europe, and circulated extensively among physicians throughout all the states of the Union. This will make the Journal a valuable advertising medium for Medical and Dental Colleges, Druggists, Surgical and Dental Instrument Makers, Gold-Beaters, File Makers, Mineral Teeth Manufacturers, and all engaged in the sale of any of the above mentioned articles.

### TERMS FOR ADVERTISING.

| | |
|---|---|
| **Fifteen lines or one-fourth of a page, single insertion in the News Sheet,** | **$5 00** |
| **Two insertions, one in the News Sheet, and one in the Journal,** | **7 50** |
| **For one year in News Sheet and Journal,** | **10 00** |

**Longer advertisements in the same proportion.**

*Spencer's Drill Stock.*—We have not only had an opportunity of examining, but also of testing the value of this instrument, and, therefore, have no hesitation in recommending it to the Dental Profession. As a piece of mechanism, it is a most ingenious contrivance, consisting of an octagonal shaft six inches in length, and five-eighths of an inch in diameter at the largest part, but the end placed in the mouth is much smaller. This shaft is hollow, and contains the movements of the instrument, which are strong and not likely to wear or become deranged. The drill, inserted at a right angle in the small end of the shaft, is made to rotate by means of a small piston rod at the other end.

*Medical Science in Spain.*—"Regular practitioners are falling very low in this country: the papers teem with advertisements, where practitioners state that no fee is required but after cure. It has likewise been announced, that the situation of medical officer to the borough of Geta (Granada) is vacant, at fifteen pence per diem." We recently saw a human mouth, which had been under treatment of a Spanish Dentist. Never in our lives did we look upon such wretched work. If the Arts and Sciences are upon a par with Medical and Surgical—Heaven save us from such charlatans.

*The Force of Habit.*—"A very striking illustration of the spirit of the times was afforded in a late sitting of the Medical Association of Paris. (This association is a new and extensive scheme, on which we shall inform our readers when the society is finally constituted.) The members were discussing different articles of the rules to be established, when it became known that fighting was going on in the streets of the capital A member immediately proposed that the debate should be adjourned, as blood was being shed in Paris; but he was opposed by M. Chassaignac, who coolly observed, that circumstances of this description should not interfere with their proceedings, as their frequency would occasion too many interruptions!"

*Annihilation of the Smell of Musk by Ergot Rye.*—Some years ago, the emulsion of bitter almonds was found to possess the property of annihilating the smell of musk, and most of the cyanic preparations evinced the same power. According to Mr. Bertot, a pharmacien of Bayeux, in Normandy, ergot of rye will produce the same effect. "I had," says he, "to prepare a certain number of pills, containing both musk and ergot; hardly were the two substances mixed than the smell completely went off, so much so, that the patient, who was not aware of the pills, only noticed the musk by the effects of flatulency.—*Jour. de Chimie. Mēdicale.*

---

*A Professionil Challenge.*—"At a recent meeting of the Academy of Medicine, Paris, much excitement was caused in this sitting by the interchange of very hostile expressions between M. Malgaigne, the reporter on the chloroform question, and M. Jules Guèrin, the editor of the *Gazette Mēdicale.* Feelings of a very inimical description have existed between these two gentlemen for several years past, and the latter seized the present opportunity, in order to attack with great animosity every one of the conclusions in M. Malgaigne's report. Matters have gone so far that a challenge was sent. One of the belligerent parties is said to have refused it, in adding the following sensible words—"A pistol-shot proves very little, and a sword-thrust nothing at all." Thus has chloroform, that somniferous agent, *par excellence,* been the means of rousing the ire of sober academicians."

---

*Medical Rank.*—Some few months since, in England, the *civil* order of the Bath was conferred on several surgeons of the Army and Navy for distinguished services. The honor was declined in every instance, on the ground that the services rendered were military and not civil. The London Lancet for August 24th, 1850, contains a proclamation that the military order of Knight Commander of the Bath (K. C. B.) has been conferred upon several eminent surgeons of the British Army and Navy. Prior to this time, this order has never been given to any medical officer whatever, and to no lineal officer below the grade of Colonel in the Army, or post-captain in the Navy. The order of Companion of the Bath (C. B.) has been given in some instances to line officers of less rank; but staff officers have been excluded until now, when medical services in the army and navy are recognized to be military services, and therefore, when distinguished, may be rewarded by the honors of Knighthood in the "most honorable Military Order of the Bath."—*Phila. North American.*

---

*A Method of Forestalling the Dangers of Chloroform.*—M. Aucelon, chief physician of the hospital of Dieuze, forwarded to the Academy of Sciences of Paris, on the 7th instant, a paper on the most frequent and least known cause of the accidents which have followed the inhalation of chloroform. The author concludes—1. That chloroform, in order to produce anæsthesia quickly, easily and safely, should never be administered except the patient be fasting. 2. That there is always a good deal of agitation and anxiety when the chloroform is inhaled by a patient whose stomach is not empty. 3. That the anæsthesia is then insufficient, and that doses are given in such cases which may endanger life. 4. That death may occur during anæsthesia if the stomach is not emptied of its contents, and freed from the pressure of the gases which fill it and clog venous circulation.

